# Enhancing MR vascular Fingerprinting with realistic microvascular geometries

**DOI:** 10.1162/imag_a_00377

**Published:** 2024-12-16

**Authors:** Aurélien Delphin, Fabien Boux, Clément Brossard, Thomas Coudert, Jan M. Warnking, Benjamin Lemasson, Emmanuel L. Barbier, Thomas Christen

**Affiliations:** Univ. Grenoble Alpes, Inserm, U1216, Grenoble Institut Neurosciences, GIN, 38000, Grenoble, France; Univ. Grenoble Alpes, Inserm, CHU Grenoble Alpes, CNRS, IRMaGe, 38000, Grenoble, France; Univ. Grenoble Alpes, Inria, CNRS, G-INP, 38000, Grenoble, France; MoGlimaging Network, HTE Program of the French Cancer Plan, Toulouse, France

**Keywords:** brain, Fingerprinting, MRI, oxygenation, tumor, vascular

## Abstract

Magnetic resonance (MR) vascular Fingerprinting proposes to use the MR Fingerprinting framework to quantitatively and simultaneously map several characteristics that emerge from vascular structure much smaller than voxel size. The initial implementation assessed the local blood oxygenation saturation (SO_2_), blood volume fraction (BVf), and vessel averaged radius (R) in humans and rodent brains using simple 2D representations of the vascular network during dictionary generation. In order to improve the results and possibly extend the approach to pathological environments and other biomarkers, we propose in this study to use 3D realistic vascular geometries in the numerical simulations. 28,000 different synthetic voxels containing vascular networks segmented from whole-brain healthy mice microscopy images were created. A Bayesian-based regression model was used for map reconstruction. We show in 8 healthy and 9 tumor-bearing rats that realistic vascular representations yield microvascular estimates in better agreement with the literature than 2D or 3D cylindrical models. Furthermore, tumoral blood oxygenation variations observed with the proposed approach are the only ones correlating with in vivo optic-fiber measurements performed in the same animals.

## 1 Introduction

Magnetic Resonance vascular Fingerprinting (MRvF) (Boux et al., 2021;Christen, Pannetier, et al., 2014;Lemasson et al., 2016) proposes to use the Magnetic Resonance Fingerprinting (MRF) (D. Ma et al., 2013) framework to quantitatively and simultaneously map several characteristics that emerge from vascular structure much smaller than voxel size. The main idea of the framework is to use a dictionary approach to estimate quantitative parameters rather than using a mathematical fit. The initial implementation of the approach used a multi-spin and gradient echo sequence acquired pre and post contrast agent injection (Ultrasmall Superparamagnetic Iron Oxide—USPIO). Acquired signals, called fingerprints, were then matched to a collection of simulated signals, called a dictionary, in order to assess the local blood oxygenation saturation (SO_2_), blood volume fraction (BVf), and average vessel radius (R) in the voxel. These microvascular properties are relevant in several brain pathologies such as cancer to monitor angiogenic processes and the presence of hypoxic tissues that resist therapies (Hompland et al., 2021;Hou et al., 2013;Sørensen & Horsman, 2020). Monitoring blood oxygenation is also valuable in the context of stroke patients to delineate the ischemic penumbra and extend the current therapeutic window (Chalet et al., 2022;Ermine et al., 2021;Zhang et al., 2020).

A major difference between MRvF and standard MRF used for tissue relaxometry is the representation of the virtual voxels during MR simulations and dictionary generation. While homogeneous voxels containing single T_1_and T_2_relaxation time values are used in MRF, vascular structures need to be considered inside the voxel for MRvF. Then, the magnetic field perturbations due to the magnetic susceptibility distributions and the phase accumulation due to water diffusion have to be computed. This leads to a considerable increase in simulation times compared to MRF simulations. A possible surrogate for blood vessels in MR simulations is the straight isotropic cylinders model (or disks in 2D), either with a single radius or with variable radii. This rather simple representation has been used extensively in mathematical models to study the blood oxygenation level dependent (BOLD) signal changes (Boxerman et al., 1995;Dickson et al., 2011;Martindale et al., 2008), as well as quantitative measurements of the vessel size index, steady-state BVf (Troprès et al., 2001), or SO_2_using the quantitative BOLD approach (Christen, Bouzat, et al., 2014;He & Yablonskiy, 2007). This representation was also used in the first MRvF paper and led to detailed maps of vascular properties in the healthy brain (Christen, Pannetier, et al., 2014). However, this simple representation of the microvasculature might not be sufficient to obtain accurate microvascular measurements in pathological environments, where vessel networks have different levels of anisotropy and where vessel shapes and tortuosity might vary drastically. While it may be difficult to include complex vessel structures in mathematical models, it should, however, be straightforward to use the MRF framework in order to include specific geometries in the signal simulations.

In recent years, several groups have used state-of-the-art microscopes and complex data processing tools to obtain whole-brain vascular networks on mice at high spatial resolution (about 1 µm isotropic). Some of these datasets are now accessible online (Di Giovanna et al., 2018;Kirst et al., 2020;Todorov et al., 2020). In addition to the availability of data, the computing power and the developments of optimized codes for MR simulations (Castillo-Passi et al., 2023;Genois et al., 2021;Pannetier et al., 2013;Weine et al., 2024) are now able to handle such large and complex datasets. A first attempt to use realistic structures in the MRvF framework was made byPouliot et al. (2017)using 6 mouse cortex angiograms stored as volumes with 1 µm isotropic resolution (total volumes were 0.27±0.05 mm^3^). Different geometrical transformations were used for data augmentation to compensate for the small number of animals and the lack of diversity of brain structures. However, the maps and quantitative results at the group level were not as promising as expected, probably due to a lack of generalization of the dictionary.

In our present study, we used 28,000 3D voxels segmented from multiple open-access datasets of whole-brain, healthy mice vascular networks as a basis to create a dictionary of signals with 3D-resolved MR simulations. Reconstruction of quantitative maps was performed using a dedicated Bayesian-based machine-learning algorithm, specifically tuned for MRvF, in order to extend the dictionary coverage to larger parameter ranges, including those expected in several pathological conditions (Boux et al., 2021). Our MRvF approach was tested in rat brains bearing tumors, and the results were compared to those obtained using 2D or 3D cylinders. MR estimates were also compared to existing histological analyses and in vivo tissue oxygenation (pO_2_) measurements made with optic fiber probes. It was found that only our proposed 3D, realistic approach produces good quality BVf, R, and SO_2_maps as well as a decreased tumoral SO_2_trend confirmed by the pO_2_measurements.

## 2 Materials and Methods

### 2.1 Generation of synthetic 3D voxels with realistic microvascular networks

Two open-access datasets of whole-brain mice vasculature were used. (1) Dataset 1 (Di Giovanna et al., 2018) (https://dataverse.harvard.edu/dataverse/mouse-brain_vasculature) contains images from a single adult male C57 mouse brain acquired with a spatial resolution of 0.65 × 0.65 × 2 µm^3^. Whole-brain images represent a volume of about 1 × 1 × 1.2 cm^3^. The available dataset is not segmented and thus needed to be processed. (2) Dataset 2 (Todorov et al., 2020) (http://DISCOtechnologies.org/VesSAP) contains already segmented blood vessels images from 9 male, 3 months old, mice brains (C57BL/6J, CD1, and BALB/c) acquired at 3 µm isotropic resolution. Due to the large volume of data represented, only two brains from the C57 mice from that second dataset were used in this work. In both datasets, images have been acquired post-mortem with a light-sheet microscope after blood vessel fluorescent staining and tissue clearing.

Both datasets were processed using ImageJ (Rasband, W.S., NIH, Maryland, USA), with the purpose of obtaining a set of MRI-sized voxels (i.e., 248 × 248 × 744 µm^3^) containing binary masks representing the vascular network. Dataset 1 was chopped into MRI-sized voxels rescaled to 2 × 2 × 2 µm^3^resolution, denoised (median and despeckle filters), segmented (tubeness plugin and thresholding), and characterized in terms of total BVf and R. The minimum ferret diameter measurement was used as the diameter of detected vessels to account for their potential in-plane orientation. Indeed, a 2D section of a vessel is a disk only if the vessel is perfectly perpendicular to the section plane. In the general case, an ellipse is observed. The minimum ferret diameter corresponds to the short axis of the detected ellipse. In 24 voxels, we compared the simulated MR signals obtained with the rescaled (2 × 2 × 2 µm^3^) and the original (0.65 × 0.65 × 2 µm^3^) spatial resolution. Results were not significantly different between the two approaches, and the coarser resolution was chosen because simulations were less computationally intense. Dataset 2 was chopped to MRI-sized voxels (248 × 248 × 744 µm^3^), with 2 × 2 × 2 µm^3^sub-voxel spatial resolution, and BVf and R were derived voxel-wise. The combination of the two datasets led to about 11,000 voxels. However, the histograms of BVf and R showed two distinct distributions of parameters, corresponding to the two different datasets. To obtain a single smooth distribution and fill-in the gaps, new MRI-sized voxels were obtained by numerical erosion of the rescaled dataset 2. A total of 28,005 MRI-sized voxels with continuous BVf and R distributions was finally obtained. Examples of voxels from dataset 1 and final histograms of BVf and R across the 28,005 voxels are shown inFigure 1.

**Fig. 1.**
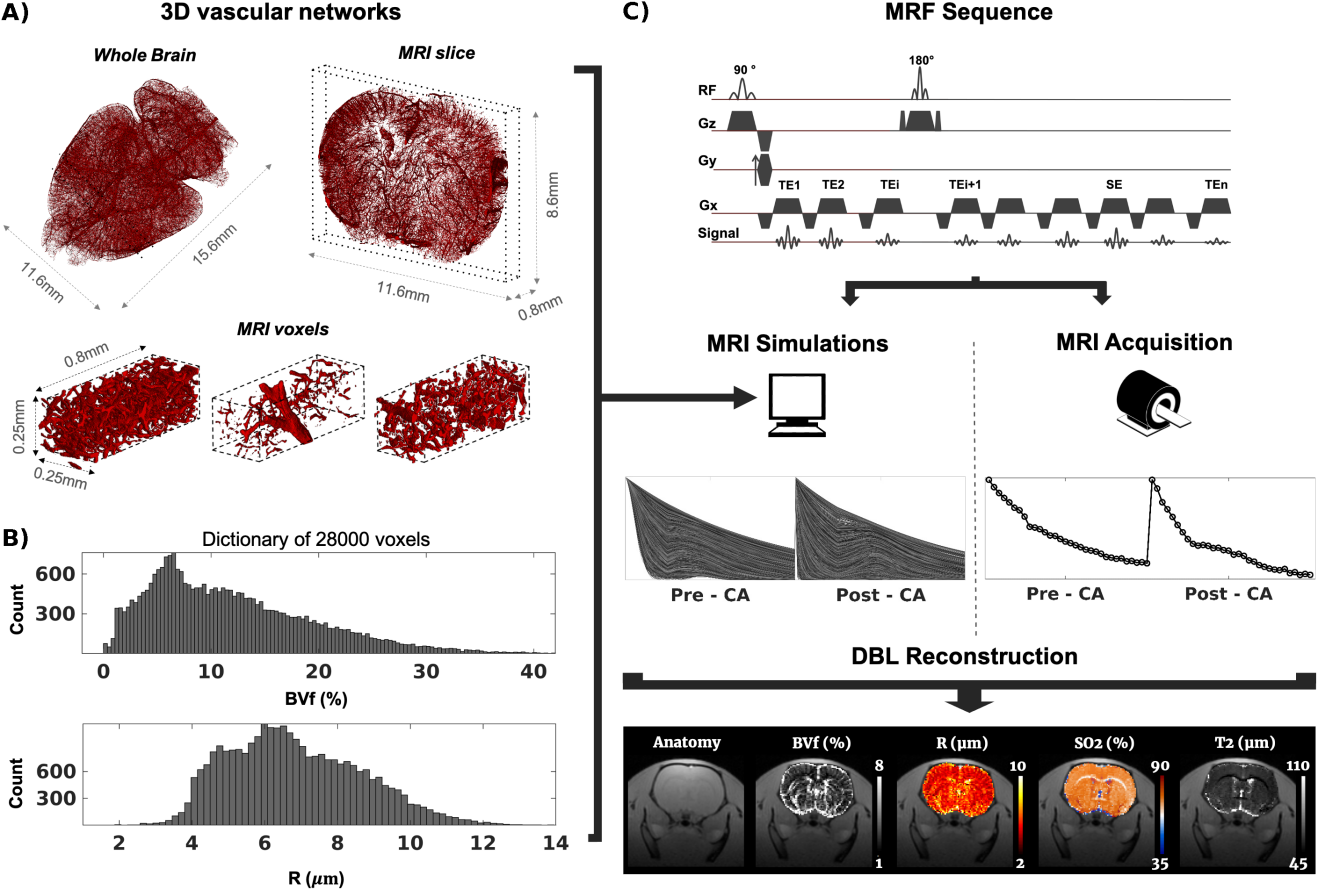
(A) Top. Examples of the whole-brain vascular network and a single 744 µm thick slice from dataset 1. Both are eroded for visibility. Bottom. Three examples of MRI-sized voxels obtained. (B) Distributions of BVf and mean radius in the 28,000 voxels generated. (C) Overview of the MRF process: a sequence is used both for numerical simulations and for in-vivo acquisitions. Parametric maps are obtained either by comparing acquisitions to simulations (DBM), or by using Dictionary-based Learning (DBL).

### 2.2 Generation of MRvF dictionaries

MR signal simulations were performed using MRVox (Pannetier et al., 2013), a Matlab (The MathWorks Inc., Natick, MA, USA) based home-made simulation tool. Briefly, for each voxel, the vascular network was given as a binary matrix. A single SO_2_value was arbitrarily attributed to the vessels in the voxel, resulting in a different magnetic susceptibility inside and outside the blood vessels. The SO_2_values were distributed according to a scrambled Sobol series and covered the value range of [35, 90]% (Boux et al., 2021). 3D magnetic field perturbations were computed using a Fourier approach (Salomir et al., 2003). In the same manner, each voxel was arbitrarily attributed a single T_2_value, again using a Sobol series, in the range of [45, 110] ms to account for transverse relaxation. Eventually, each voxel is thus associated with 4 parameters: BVf, R (both imposed by the vascular network considered in the voxel), SO_2_and T_2_. Water diffusion effects were taken into account using a diffusion kernel convoluted with the magnetization matrix (Bandettini & Wong, 1995;Klassen & Menon, 2007). The water diffusion coefficient was set to 1,000 µm^2^/s. Simulations were performed using a main magnetic field of 4.7T. The response of each voxel to the GESFIDSE sequence is simulated pre- and post-injection of a contrast agent, see section “MR data acquisition” for more details.

**Fig. 2.**
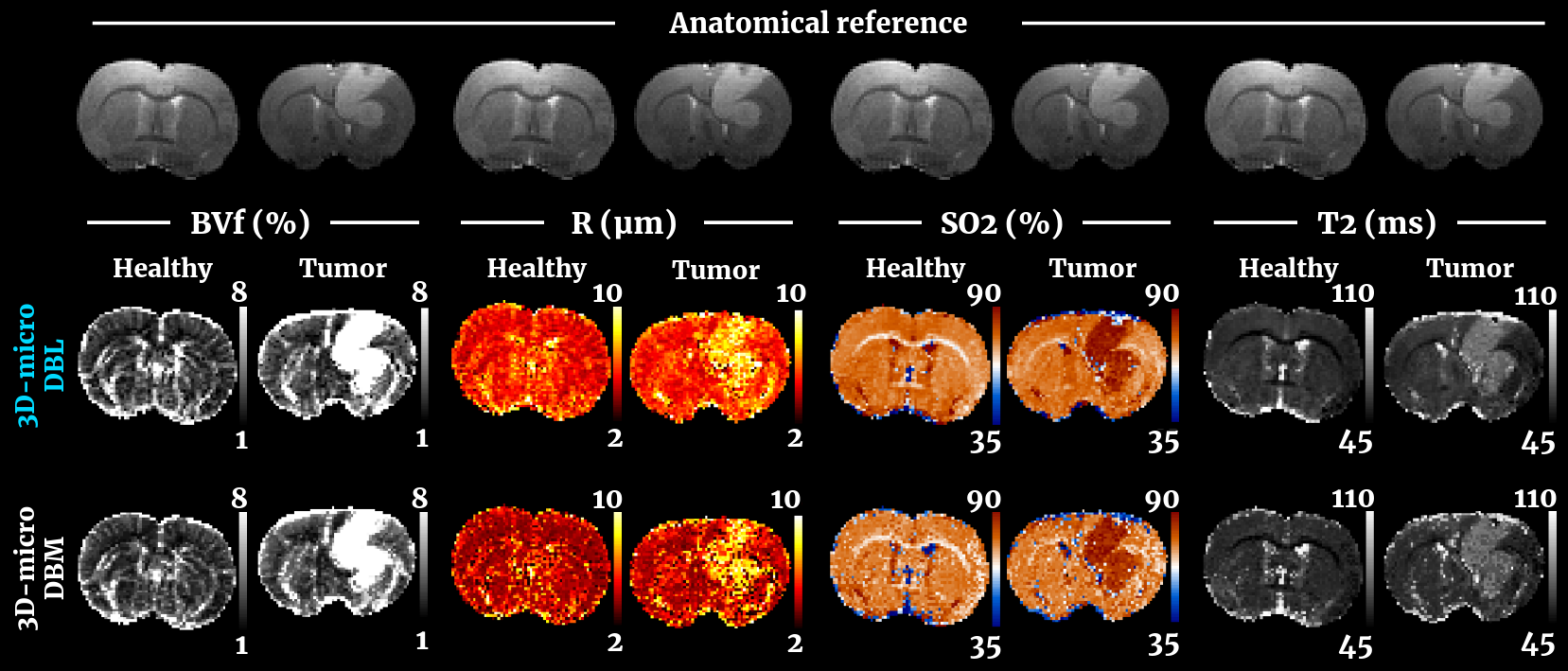
Comparison of parametric maps obtained through MRvF using the 3D-micro dictionary for healthy and tumoral tissue. For each of the 4 parameters, the top and bottom lines correspond to the result from dictionary-based learning (DBL), and dictionary-based matching (DBM), respectively. Values outside the presented range are clipped to the corresponding extrema.

Three dictionaries were eventually generated. The “3D-micro” dictionary is based on the 3D realistic voxels obtained using the microvascular data described above. The vascular network resolution is that of the segmented voxels, that is, 2 µm isotropically. The “2D-synth” dictionary is based on 2D voxels, as described in the initial MRvF implementation (Christen, Pannetier, et al., 2014;Lemasson et al., 2016), namely vessels are represented as disks in the plane (248 × 248 µm^2^), with a fixed radius. The “3D-synth” dictionary is based on 3D voxels containing straight cylinders with variable radii. The voxel size is the same as for the 3D-micro case: 248 × 248 × 744 µm^3^. The cylinders are isotropically oriented in the volume. Both 2D-synth and 3D-synth dictionaries were generated with BVf, R, SO_2_, and T_2_combinations similar to those obtained in the 3D-micro dictionary. Each dictionary thus contains between 28,002 and 28,005 entries, the number varying slightly as the geometrical constraints of the cylinder generation cannot always accommodate all (BVf, R) combinations. Both synthetic dictionaries use voxel networks with a 1.94 µm isotropic resolution. Examples of the three types of voxels are given inSupplementary Figure 1. The coverage of the vascular parameter space by each of the 3 dictionaries is similar and shown inSupplementary Figure 2.

### 2.3 MR data acquisition

All procedures were reviewed and approved by the local ethics committee (Comité éthique du GIN n°004) and were performed under permits 380820 and A3851610008 (for experimental and animal care facilities) from the French Ministry of Agriculture (Articles R214–117 to R214–127 published on February 7, 2013), and reported in compliance with the ARRIVE guidelines (Animal Research: Reporting in Vivo Experiments). All surgery was performed under lidocaine/isoflurane anesthesia, and proper efforts were made to minimize suffering.

MRI data acquisition was conducted on a horizontal 4.7T Bruker (Bruker Biospin, Ettlingen, Germany; IRMaGe facility) system. Two groups of animals (n = 17 total) were scanned: (1) 8 healthy Wistar rats (7 weeks old, 268±23 g, Charles River, France) were used as controls. (2) 9 Fischer 344 rats (7 weeks old, 235±13 g, Charles River, France) implanted with 9L (9LGS, ATCC, American Type Culture Collection) tumors were imaged 14 to 16 days after induction. Anesthesia was induced by the inhalation of 5% isoflurane (Abbott Scandinavia AB, Solna, Sweden) in an 80% air–20% O_2_mix and maintained throughout the measurements with 2–2.5% isoflurane through a facial mask. The imaging protocol included relaxometry (T_2_- using a multi-echo sequence - and${\text{T}}_{2}^{\text{*}}$- using a multi-gradient-echo sequence), Apparent Diffusion Coefficient (ADC) (3 orthogonal directions), and perfusion acquisitions, as described inLemasson et al. (2016). Vascular fingerprints were acquired using a 2D Gradient-Echo Sampling of the Free Induction Decay and Spin Echo (GESFIDSE) (J. Ma & Wehrli, 1996) sequence, TR = 4,000 ms, 32 echoes,ΔTE = 3.3 ms, SE = 60 ms, NEX 1, 5 slices, 128 × 128 × 32 matrix, and 234 × 234 × 800 µm^3^resolution. One GESFIDSE acquisition was performed before and one after injection of ultrasmall superparamagnetic iron oxide (USPIO) (P904, Guerbet, France, 200 µmol Fe/kg), and the two acquisitions were concatenated in order to produce a single fingerprint per voxel.

### 2.4 MR data processing

All processing was performed on MP3 (Brossard et al., 2020), a Matlab-based image processing software. Three processing approaches were considered: two dictionary-based MRvF reconstructions: dictionary-based matching and dictionary-based learning, and the original analytical approach (Christen, Bouzat, et al., 2014;Troprès et al., 2001).

For MRvF processing, two reconstruction methods were used: (1) In the Dictionary-based matching (DBM) approach, the dot product of each acquired fingerprint with the whole dictionary was computed. The dictionary entry yielding the highest value was kept as the best match, and the corresponding parameters (BVf, R, SO_2_, T_2_) were used to create the parametric maps. (2) In the Dictionary-based learning (DBL) approach, a Bayesian-based method (Boux et al., 2021) was used to learn the relationship between the signals and the parameters space. Once trained, the algorithm produces BVf, R, SO_2_, and T_2_values in response to the acquired fingerprints. Estimates coming from the DBL method can fall outside of physical ranges. In that case, BVf and SO_2_values were clipped between 0% and 100%, that is, values exceeding the upper (or lower) limit were set to that limit, and R values were clipped between 0 and 250 µm. T_2_values were only clipped to 0 ms on the lower end. When visualizing maps, displayed values have different clips for better visual comparison between the different methods. An overview of the whole process in one animal is presented inFigure 1.

Microvascular properties were also calculated according to previously published methods based on analytical models. T_2_and${\text{T}}_{2}^{\text{*}}$maps obtained pre and post contrast agent (CA) injection (Christen, Bouzat, et al., 2014) were combined with the ADC map to compute the local BVf, SO_2_, and Vessel Size Index (VSI) (an average weighted by higher values of the vessel radius whose value may be compared to R). Note that the analytical models, therefore, use more data than the MRvF which does not require T_2_,${\text{T}}_{2}^{\text{*}}$, and ADC maps.

### 2.5 In vivo pO _2_ measurements

In order to obtain reference oxygenation values in our animals, pO_2_measurements were made in the tumor group after the imaging protocol using optic fiber probes (Oxylite, Oxford Optronix, Oxford, UK) and under the same anesthesia as the one described above. The pO_2_and SO_2_values are related by the monotonic hemoglobin dissociation curve; an increased pO_2_is thus linked to an increased SO_2_. Using guidance from MR anatomical images, two catheters were implanted to guide the optical fibers in both the tumoral and contralateral hemispheres. Measurements were repeated between 5 and 10 times, and at two different depths 1.5 mm apart. In total, pO_2_estimates were available in 6 animals, as the measurements failed in one rat and two others died before this step. These measurements are performed in a volume surrounding the tip of the probe which is much larger than an MRI voxel. Direct correlation between OxyLite pO_2_and MRvF SO_2_will, thus, be impossible. Yet, we expect the monotonous relation between pO_2_and SO_2_to allow us to confirm oxygenation variations between the tumor and the contralateral hemisphere.

### 2.6 Numerical and statistical analysis

Regions of interest (ROI) were manually drawn using MP3. For the control group, an ROI called “Healthy” was drawn in the striatum of the left hemisphere. For the tumoral group, lesions were manually delineated from anatomical and ADC scans (“Tumor” ROI), and contralateral ROIs (“Contra”) were drawn in the other hemisphere, trying to match both tumor location and volume. We carefully avoided including ventricles in these ROIs. For the 4 parameters, voxel values were averaged inside each ROI, for each animal. Statistical significance between methods was evaluated through a 2-sample t-test with a 0.05 p-value threshold.

## 3 Results

### 3.1 3D-micro DBL versus DBM

Figure 2(top row) shows the results obtained in one representative animal of each group (control and tumor) using the 3D-micro dictionary and the DBL reconstruction. The tumor-bearing animal was chosen for its well defined, not too big lesion that allows for a good contrast. The healthy animal was selected at random. Qualitatively, it can be observed that without the need of any prior information on the tissue composition, the MRvF method produces high-quality BVf maps exhibiting fine details of the vasculature while flatter contrasts are obtained for R or SO_2_maps.Supplementary Figure 3compares a BVf map with an image from the post-CA injection GESFIDSE, on which large vessels appear darker. T_2_maps show the expected contrasts, with higher values in the CSF. Averaged values of approximately BVf = 3%, R = 5 µm, SO_2_= 75%, and T_2_= 55 ms in healthy tissues are in line with previous literature reports and will be discussed below. In the tumor-bearing animal, the lesion is clearly visible in all parametric maps with a global increase of BVf, heterogeneous R variations and a global increase of SO_2_indicating hyperoxic areas.

The same maps obtained in the same animals, using the same 3D-micro dictionary, but analyzed using the standard matching approach (DBM) are shown inFigure 2(bottom row). The BVf maps obtained through DBL tend to exhibit a better contrast for large blood vessels and are less noisy. For both methods the tumor is visible. The SO_2_maps obtained with DBL in healthy tissues present slightly lower values than with DBM and although the tumor is visible in both cases, the contrast is higher with DBL. Finally, the T_2_maps obtained with DBL are smoother with comparable values in both healthy tissue and lesion. These differences can be explained by the fact that the DBL method is able to interpolate parameter values for which signals were not simulated. In the rest of this article, only results obtained with DBL will be discussed. Yet, the numerical analysis of the DBM results is provided in Supplementary Material (Supplementary Figure 4).

### 3.2 3D-micro versus synthetic dictionaries

We illustrate inFigure 3the results obtained in two other animals from both groups. The animals were selected on the same criteria as before. Here, the maps obtained with the 3D realistic model and DBL reconstruction are compared to those obtained with the cylindrical model (2D/3D simulations and analytical models), in order to assess the benefits of using realistic vascular geometries. Quantitative results obtained for all groups are presented inFigure 4using boxplot representations. All the results from crossed statistical analyses between all methods and all ROIs are summarized inSupplementary Tables 1–4. The quantitative data analysis performed for DBL onFigure 4was also performed for DBM (see previous section) and can be found inSupplementary Figure 4.

**Fig. 3.**
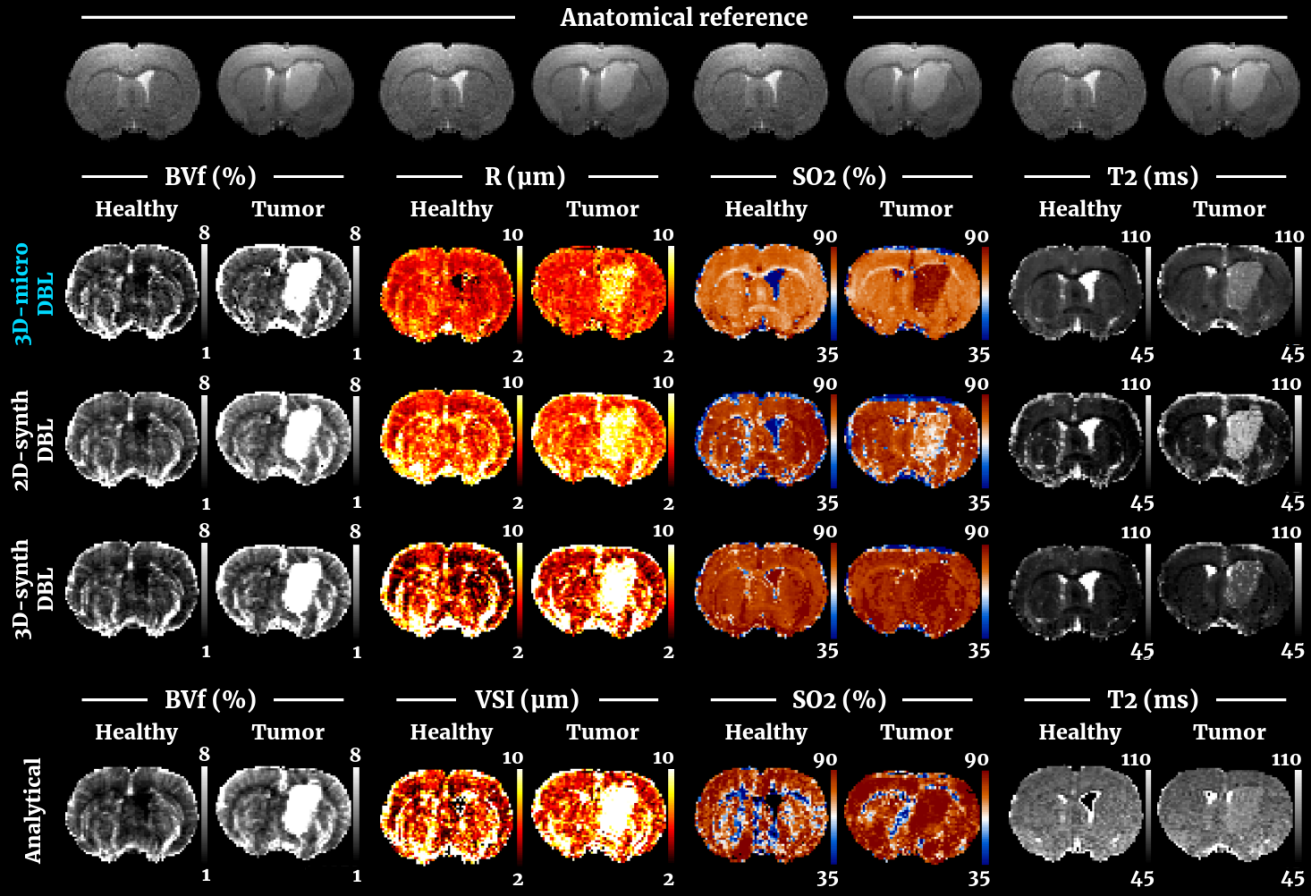
Comparison of parametric maps obtained through MRvF using the DBL reconstruction and the 3D-micro dictionary. Healthy and tumoral brains are presented for each parameter column. The top three rows correspond to MRvF results with the 3D-micro, the 2D-synth, and the 3D-synth dictionaries, respectively. The bottom row corresponds to the vascular parameters obtained with the analytical approach.

**Fig. 4.**
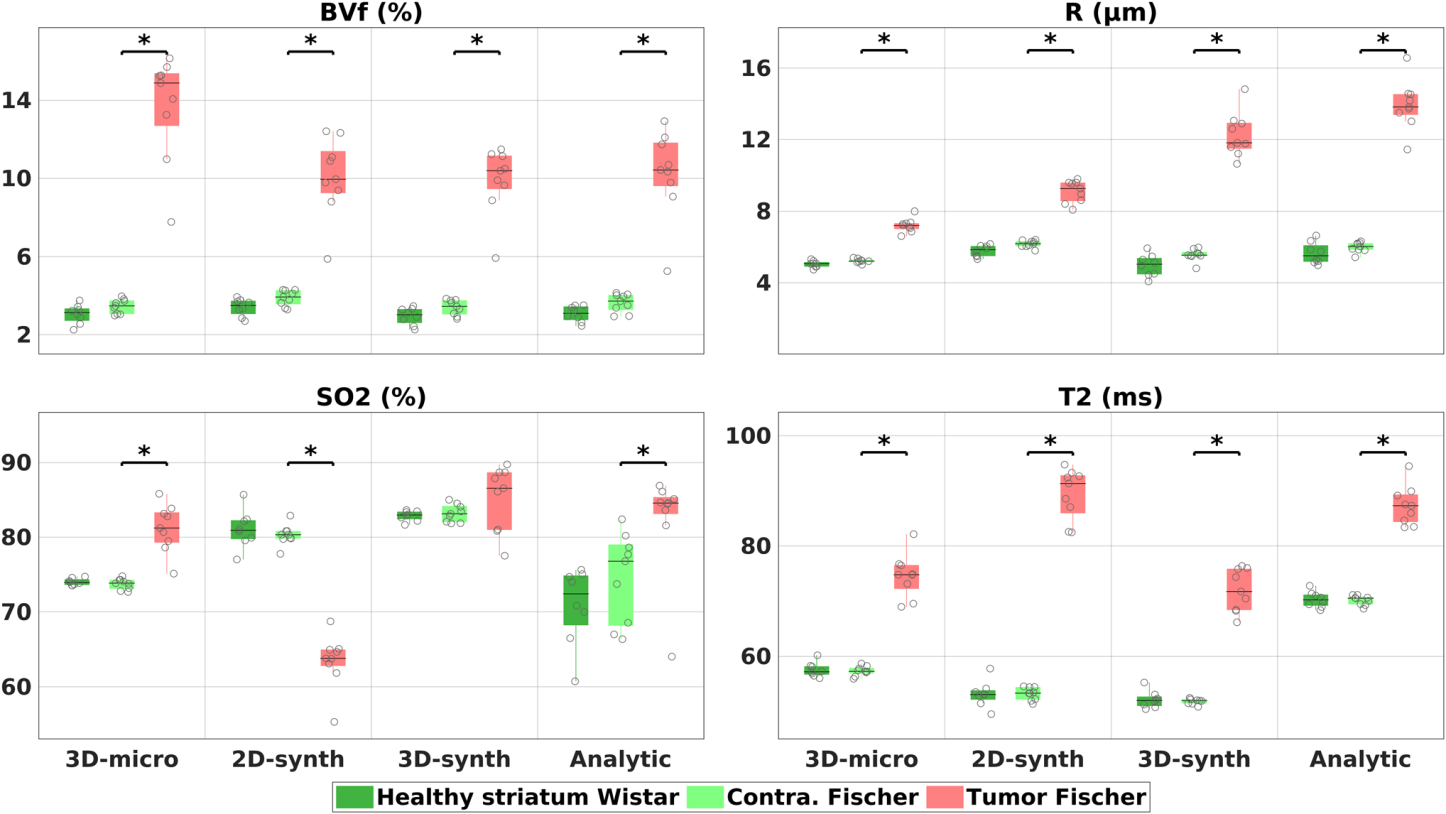
Quantitative estimates of the 4 parameters for the different methods. All results from MRvF were obtained with the DBL method. “Healthy” values are averaged in the striatum for each animal, “Tumor” values from the lesion, and “Contra.” from a contralateral ROI matching the tumor’s location and size. Stars indicate the significance with a p-value≤0.05.

For BVf estimates (Figs. 3and4top left panel), all methods produced maps with visible vascular structures. No significant differences were found between the approaches in the healthy tissues (averages between 3.0±0.41% and 3.9±0.42%). All maps show visible tumors, with a statistically significant increase of BVf values in the lesion for all methods (from 6.2±1.7% to 10.3±2.4% average increase). The values found in the tumor with 3D-micro were, however, higher than with the cylinder approaches (cfSupplementary Table 1).

For R estimates (Figs. 3and4top right panel), the 3D-micro dictionary yielded maps that differed from the ones obtained with the 2D-synth dictionary. The healthy tissues were also less contrasted, which was confirmed in the lower dispersion of mean values inFigure 4. Mean values obtained with the 3D-micro approach were 5.0±0.2 and 5.2±0.1 µm in ROI Healthy and Contra, respectively. The 2D-synth and analytical maps produced averaged values between 5.7±0.6 and 6.2±0.2 µm. The tumor is visible with all methods, but the difference between non-lesion and lesion tissues is less pronounced when using the 3D-micro dictionary. Indeed, we measured an averaged significant difference of 2.0±0.37 µm between the Contra and Tumor ROIs, while we found an increase from 2.9±0.6 to 7.9±1.3 µm with the other methods.

For SO_2_estimates (Figs. 3and4bottom left panel), the 3D-micro dictionary also produced results that differed from the other methods. Normal-appearing tissues presented lower average SO_2_values (74.0±0.4% and 73.7±0.7%, Healthy and Contra ROIs, respectively) than those obtained through MRvF with the other dictionaries (between 80.3±1.3% and 83.2±1.1%). The analytic method produced maps with a mean value in the healthy tissues comparable to that obtained with the 3D-micro dictionary, but the maps are noisy. Conversely, the 3D-micro maps are flatter. The contrast between normal-appearing tissues and tumors is clearly pronounced in the 3D-micro maps.Figure 4andSupplementary Table 3show that only the 3D-micro dictionary and the analytical methods found a significant SO_2_increase in the lesion (average increase of 7.5±3.0% and 7.7±7.1%, respectively). On the contrary, the 2D-synth dictionary showed hypoxia in the Tumor ROI, while the 3D-synth dictionary yielded no significant difference.

For T_2_estimates (Figs. 3and4bottom right panel), the MRvF maps have comparable contrasts. The T_2_maps obtained from the multi-echo sequence (analytical method) appear noisier and flatter than the T_2_maps obtained with the MRvF approach based on only one spin echo. Moreover, the analytical approach failed to provide T_2_estimates in one ventricle, whereas all MRvF approaches yielded a T_2_estimate in that area. The values estimated in the normal-appearing tissues were higher (70.3±1.4 – 70.1±0.8 ms, Healthy and Contra ROIs respectively) than for the MRvF methods (between 51.9±0.5 and 57.6±1.2 ms). The 3D-micro dictionary yielded values of 57.6±1.2 and 57.3±0.8 ms in the Healthy and Contra ROIS, while the 2D- and 3D-synth dictionaries produced values in the range of 51.9±0.5 to 53.2±1.1 ms. In all cases, the tumor was visible and significantly distinct from the contralateral tissue. The variation seen in the lesion, compared to healthy tissues, with the 3D-micro dictionary and the analytical method were comparable, with an increase of 17.2±3.4 and 17.2±3.2 ms, respectively. On the other hand, the 3D-synth and the 2D-synth yielded an increase of 20.1±3.5 and 36.2±4.1 ms, respectively.

### 3.3 Oxylite pO _2_ measurements

**Fig. 5.**
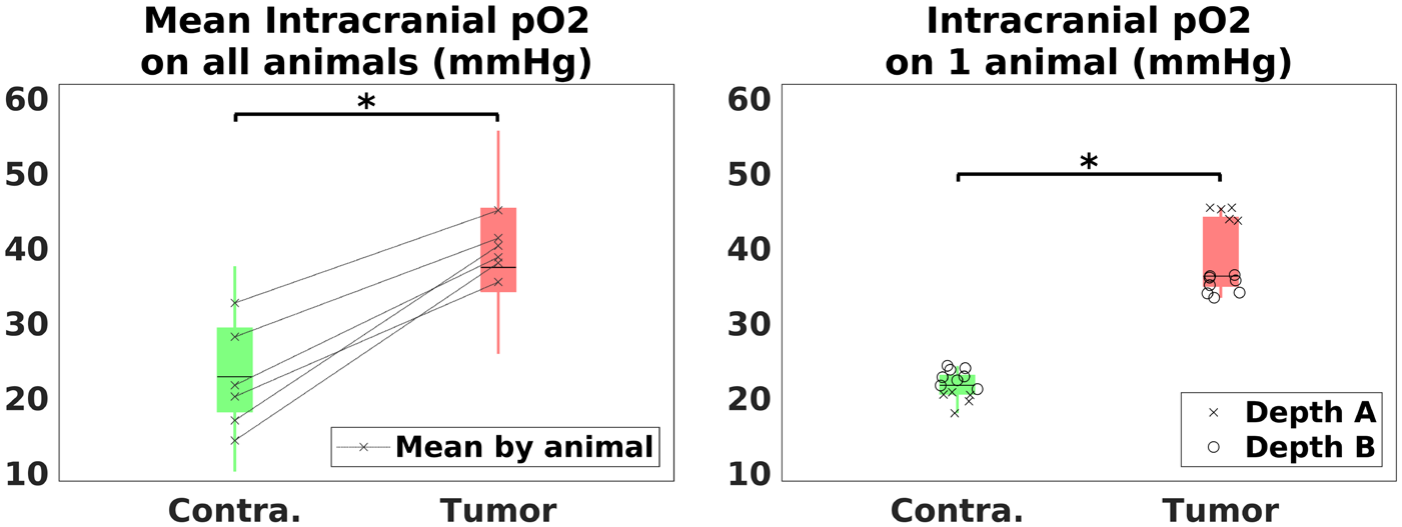
pO_2_measurements obtained through Oxylite measurements. Six animals were implanted with optic fiber measuring the pO_2_at two different depths, both in the lesion and the contralateral hemisphere. Between 5 and 10 measurements were performed at each depth. (left) Boxplots of the values obtained in both zones on all animals. The mean value in each hemisphere for each animal is shown. (right) Measurements obtained in one animal. Boxplots and actual values, at two different depths, are shown. Stars indicate the significance with a p-value ≤ 0.05, for a 2-sample t-test.

Figure 5presents the pO_2_measurements. The left panel shows boxplots of the values obtained in the contralateral and tumor tissues in all the animals, combining the results from the two probed depths. The mean value in each of the considered animals is also shown. The right panel shows the values obtained in the tumor animal presented onFigure 3. The single readings at each depth are given, as well as the boxplots corresponding to each hemisphere. These values clearly indicate a pO_2_increase in the tumor in all animals, confirming the trend observed with the 3D-micro dictionary and the analytical method.

## 4 Discussion

We show in this paper the possibility to include realistic vascular networks extracted from high-resolution whole-brain microscopy acquisitions into the MRvF framework. Using approximately 28,000 synthetic voxels to create the dictionary, we obtained encouraging MRvF microvascular maps in rat brains. In particular, the BVf maps exhibit fine details and quantitative values in the range of those previously reported using MRI perfusion techniques. It is worth mentioning that the results obtained using fewer voxels in the dictionary (<12,000) led to poor and unreliable results (data not shown). This is consistent with the difficulties already identified byPouliot et al. (2017)working with a small number of realistic voxels, and noting that the MRvF signal pattern can vary between voxels with similar vascular parameters.

It is also important to note that the method performs well on rat data, although the dictionaries are based on mice microvasculature. A high similarity of vascular networks can be expected between mice and rats. Capillary networks are expected to be similar across mammalians:Smith et al. (2019)showed that, at least for vessel length, mice and humans capillary networks are similar except for a scaling factor. More specifically,Unekawa et al. (2010)measured similar red blood cells velocities in rats and mice capillaries, as well as similar capillary length, hinting at very similar capillary networks. The good performance of our proposed method on rat brains despite the use of mice datasets was thus to be expected. In the same fashion, only healthy vasculature was considered. Different anatomical regions are well visible without the need of any prior information, nor specific tissue targeting during dictionary generation (e.g., specific T_1_and T_2_values for WM, GM, and CSF). The higher myelin fraction in WM was not simulated and is interpreted during the reconstruction step as a decreased SO_2_in the corpus callosum, due to a magnetic susceptibility source absent from the simulated signals. It is likely that using species-specific vascular network representation and abnormal networks from pathological lesions, as well as including more tissue characteristics, would further improve the results.

One of the main purposes for using realistic vascular networks in MRvF is to improve the quality and precision of the maps initially obtained using the cylinder models. In particular, it was hoped that realistic simulations that include blood vessels with different shapes, densities, or anisotropic orientations could capture the inherent heterogeneity of the microvascular networks. Our comparison of the different simulation approaches seems to validate this hypothesis. In healthy tissues, we found BVf estimates close to 3% with the 3D-micro dictionary. This is consistent with previous histological measurements made in similar animals (Lemasson et al., 2013), and significantly lower than BVf values found here with the 2D cylinders model (≈ 3.5%). The R values found with the 3D-micro dictionary (≈5 µm) were also slightly closer to histology (≈ 5 µm;Lemasson et al., 2013) than the 2D-synth dictionary (≈ 6 µm). The 3D cylinder models provided BVf and R results closer to the reference values than the 2D models but were often significantly different from the 3D-micro estimates. We also found significant differences between 3D-micro and cylinders approaches for the SO_2_estimates. In healthy rat brain, oxygenation measurements using^15^O PET technology,^17^MRI, or near-infrared spectroscopy suggest that SO_2_should be homogeneous and range from 55% to 75% in healthy rat brains (Hashem et al., 2020;Horitsugi et al., 2017;Zhu et al., 2013). These observations are in line with the 3D-micro estimates (SO_2_≈ 74% vs.>80% for cylinder approaches). SO_2_values in orthotopic 9L tumors are not well established in the literature. However,^18^F-FMISO PET studies indicate that the tumor is not hypoxic (Stokes et al., 2016;Valable et al., 2017). In a similar fashion, pimonidazole stainings of ex-vivo orthotopic 9L tumoral tissue are negative (Valable et al., 2017). Note that this is not the case for ectopic 9L tumors, which are sometimes used as a model of hypoxic tumors (Hou et al., 2013). Electron Paramagnetic Resonance (EPR)-based pO_2_measurements in orthotopic 9L tumors 14 days after cell implantation are slightly higher than in the contralateral hemisphere (37±7 mmHg, vs. 35±4 mmHg, resp.) (Khan et al., 2012). This agrees with our own OxyLite pO_2_measurements (40.0±3.3 mmHg) which are higher in the tumor than in the contralateral hemisphere. It is especially interesting to note that a study conducted on healthy Fischer rat brains found that an EPR-based pO_2_of 35.75±2.77 mmHg corresponds to an OxyLite measurement of 24.5±5.75 mmHg (O’Hara et al., 2005). This last value again agrees with our own OxyLite measurements (22.5±7.0 mmHg). The decreased tumoral SO_2_estimated with the 2D-synth dictionary thus appears erroneous, while the elevated SO_2_found with the 3D-micro dictionary and the analytical method are in agreement with our Oxylite pO_2_measurements. It is worth noting that the analytical method requires several additional scans and thus an increased scan time. For R measurements, the 3D-micro values found in the tumor were statistically different from the ones found in healthy tissues but the difference was less pronounced than with the other methods. A reason for this discrepancy could be the absence of a tumoral network in the realistic dictionary that is not entirely compensated by the learning approach. A study that would include pathological networks in the dictionary as well as tests in other types of pathologies could lead to a better understanding of these results. This increased vessel radius is supported by PET findings of an elevated perfusion in orthotopic 9L tumors (Stokes et al., 2016). Finally, T_2_values found with the MRvF approach in both healthy tissues and tumoral environment seem to agree with previous reports using standard relaxometry methods (Vonarbourg et al., 2004). However, our multi-spin echo sequence and analytical analysis provided higher values than the MRvF approach. This variation could be ascribed to different factors. GESFIDSE signals are minimally impacted by water diffusion (hence reasonable quantitative estimates using a dictionary with a single ADC value), but still more than multi-spin echo signals. Stimulated echoes are present in the multi-spin echo sequence but not in the GESFIDSE (one spin echo, sampled with 32 gradient echoes). A dependence on the slice-selection pulse profile is also expected. A further exploration of this difference is certainly of interest as it may carry additional information about the voxel properties. For this purpose, one could try and use the MRF framework to process the multi-spin echo acquisition, as done inBen-Eliezer et al. (2015).

A second purpose for using realistic networks in MRvF is the possibility to extract more information from the acquired fingerprints. In our study, we have focused the analysis on the measurements of BVf, R, and SO_2_values. It is likely that the encoding capacity of the sequence used would be too limited to measure more parameters. Yet, given the right sequence with the right sensibilities, several other parameters of the vascular networks should be accessible. For example, tortuosity, global anisotropy, fractal dimension (Chen et al., 2018;Forster et al., 2017), or distribution of oxygenation are also expected to impact the MR signal. For the moment, these parameters act as confounding factors in our MRF analysis but could be measured if taken properly into account and added as extra dimensions in the dictionaries. By using more complex geometries, it should also become possible to measure dynamic biomarkers such as blood flow or distribution of transit times using MRvF. Indeed, several studies have shown that combinations of angiographic data with vascular growth algorithms can generate entire synthetic brain microvasculature with closed networks and realistic properties (Cury et al., 2021;Hartung et al., 2021;Linninger et al., 2019;Talou et al., 2021). It should then be possible to perform simulations of blood flow, pO_2_, and SO_2_distributions and to compare the results to MRF acquisitions.Supplementary Figures 5 and 6show that the impact of a distributed oxygenation across the vessel network has a limited effect on signals obtained with the GESFIDSE sequence, compared to a single SO2 value per voxel. We conclude that although this approximation might be a small confound in the current approach, it might also be a good opportunity to glean further information in future studies. Realistic simulations could also be used within the MRF ASL technique (Fan et al., 2024;Su et al., 2017) or to follow the bolus of exogenous contrast agents.

Increasing the number of dimensions in the dictionaries and the complexity of the simulations automatically impact the simulations and reconstruction times. In order to keep reasonable processing times, we used a combination of a machine-learning algorithm with a Sobol approach to sample the parameter space. This allows, for future studies, the addition of new dictionary dimensions, such as the diffusion coefficient, without increasing the size exponentially. In the current state, working on a computing server with 104 CPU cores divided in 13 groups of 8 cores each, simulating a GESFIDSE sequence on a single 3D voxel requires about 10 seconds. In order to further accelerate the process and be able to create a large variety of realistic MRF voxels, simulations could be performed with GPU implementations (Castillo-Passi et al., 2023;Wang et al., 2020;Weine et al., 2024) or to create surrogate deep learning simulators (Huang et al., 2023;Liu et al., 2021;Yang et al., 2020).

Finally, the work presented here focused on the reconstruction part of the MRF framework, and it is clear that complex reconstruction will only be useful if linked to an efficient MRF sequence design. In this paper, we used the GESFIDSE sequence which consists of a single spin echo experiment, sampled on 32 time points with gradient echoes. State-of-the-art MRF literature generally considers multi-GE sequences, such as FISP of bSSFP, with hundreds or thousands of time points (Fan et al., 2024;Jordan et al., 2021;Marty et al., 2023). More complex sequences are thus investigated to find a pattern that would retain a similar or increased sensitivity under high acceleration factors, taking advantage of sparse, non-cartesian acquisition schemes (Coudert et al., 2022;Delphin et al., 2020,^2021^). A number of studies have already shown great improvements in relaxometry measurements when using automatic MRF sequence optimization algorithms (Guenthner et al., 2023;Heesterbeek et al., 2023;Jordan et al., 2021;McGivney et al., 2020). These tools could be applied here to further improve the results, reduce the acquisition time, and even remove the need for an exogenous contrast injection.

## Supplementary Material

Supplementary Material

## Data Availability

MP3 and the 2D version of MRVox are open-source software available here:https://github.com/nifm-gin. The 3D version of MRVox can be made available after discussion with the corresponding author. Animal data and dictionaries can be made available upon reasonable request to the corresponding author.
