# Supplementary material for: Enhancing MR vascular Fingerprinting with realistic microvascular geometries
Source: Imaging Neurosci (Camb). 2024 Dec 16;2:imag-2-00377. doi: 10.1162/imag_a_00377 (PMC12315760; doi:10.1162/imag_a_00377)
Supplement: Supplementary Material [file imag_a_00377-supp.pdf]

Enhancing MR Vascular Fingerprinting with realistic microvascular geometries

Aurélien Delphin,[1,2] Fabien Boux,[1] Clément Brossard, [1] Thomas Coudert, [2]
Jan M. Warnking, [2] Emmanuel L. Barbier, [2] Thomas Christen[2*]

[1]Univ. Grenoble Alpes, Inserm, U1216, Grenoble Institut Neurosciences, GIN, 38000, Grenoble, France

[2]Univ. Grenoble Alpes, Inserm, CHU Grenoble Alpes, CNRS, IRMaGe, 38000, Grenoble, France

[3]Univ. Grenoble Alpes, Inria, CNRS, G-INP, 38000, Grenoble, France

[4]MoGlimaging Network, HTE Program of the French Cancer Plan, Toulouse, France

*Correspondence: thomas.christen@univ-grenoble-alpes.fr

SUPPLEMENTARY MATERIAL

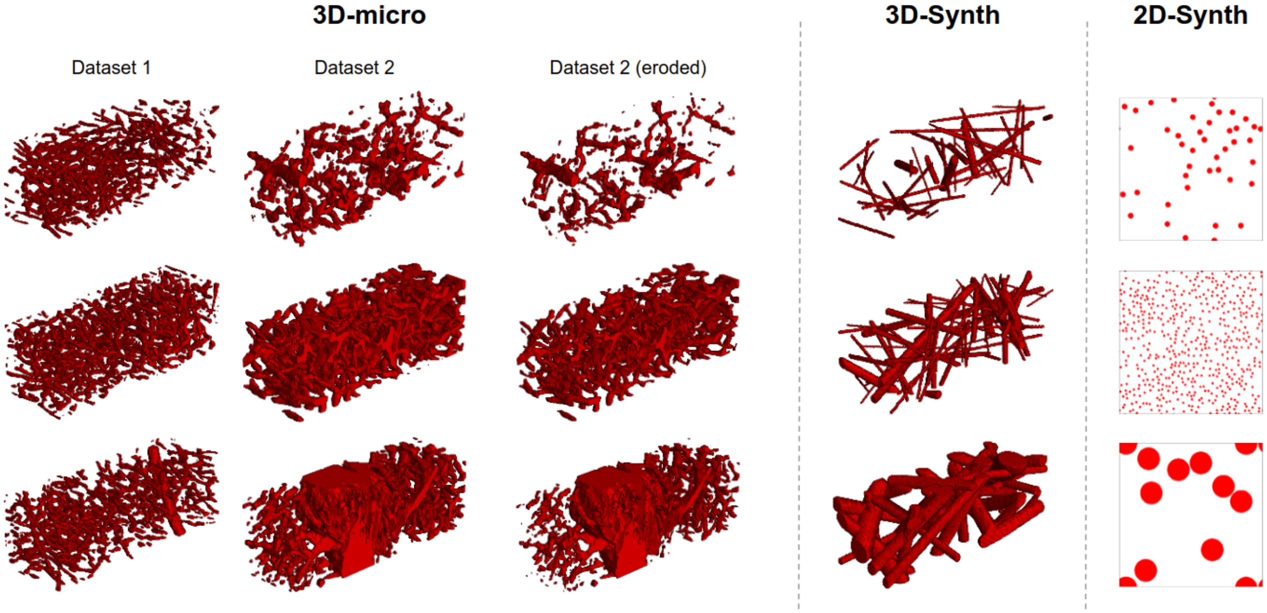

Sup.Fig. 1: Examples of voxels (248x248x744 μm^3) or pixels (248x248 μm^2) considered in each dictionary.

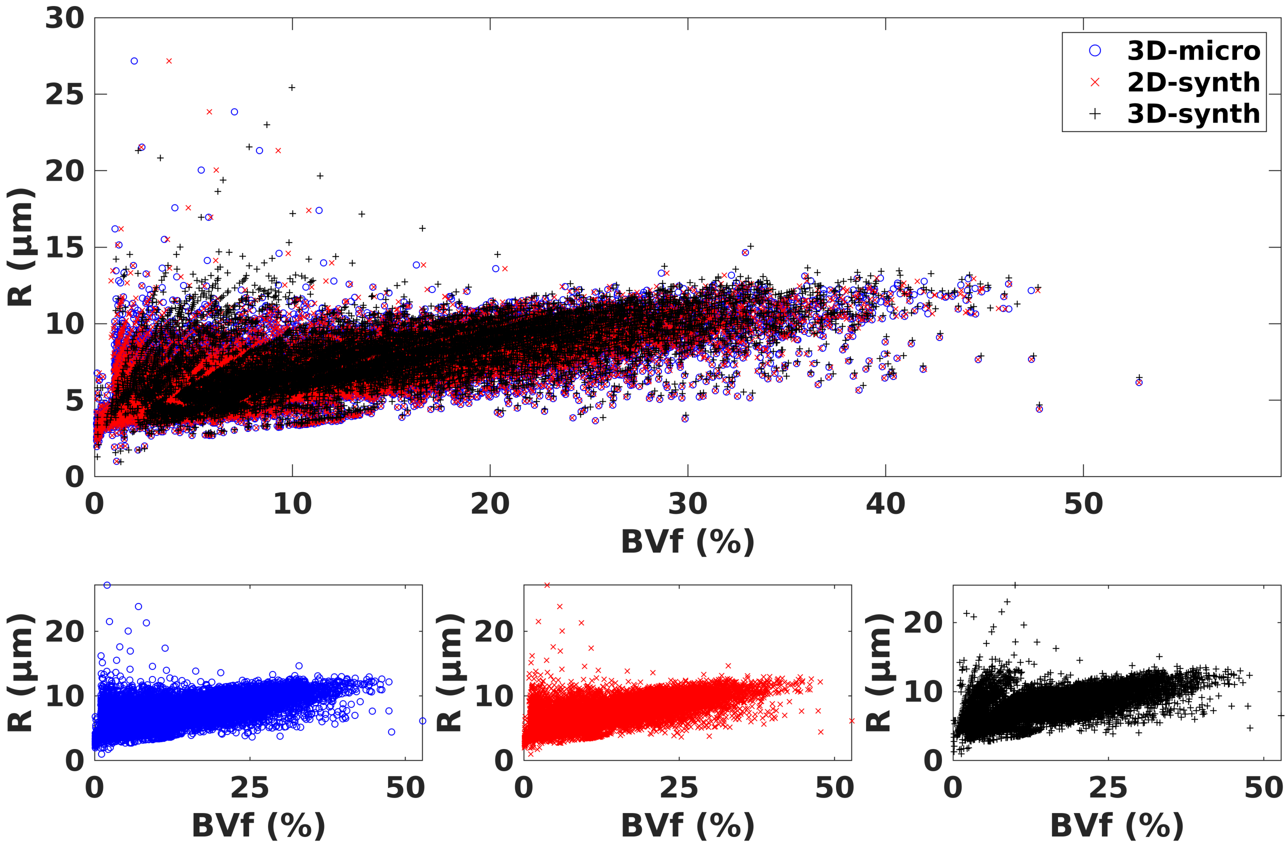

Sup. Fig. 2: (BVf, R) parameter space coverage for the three dictionaries generated. Top panel shows a superposition of the 3 lower panels, each corresponding to a dictionary. Discrepancies between the microscopy-based results and the synthetic ones come from the geometrical impossibility of our generation methods to accommodate every BVf and R combination.

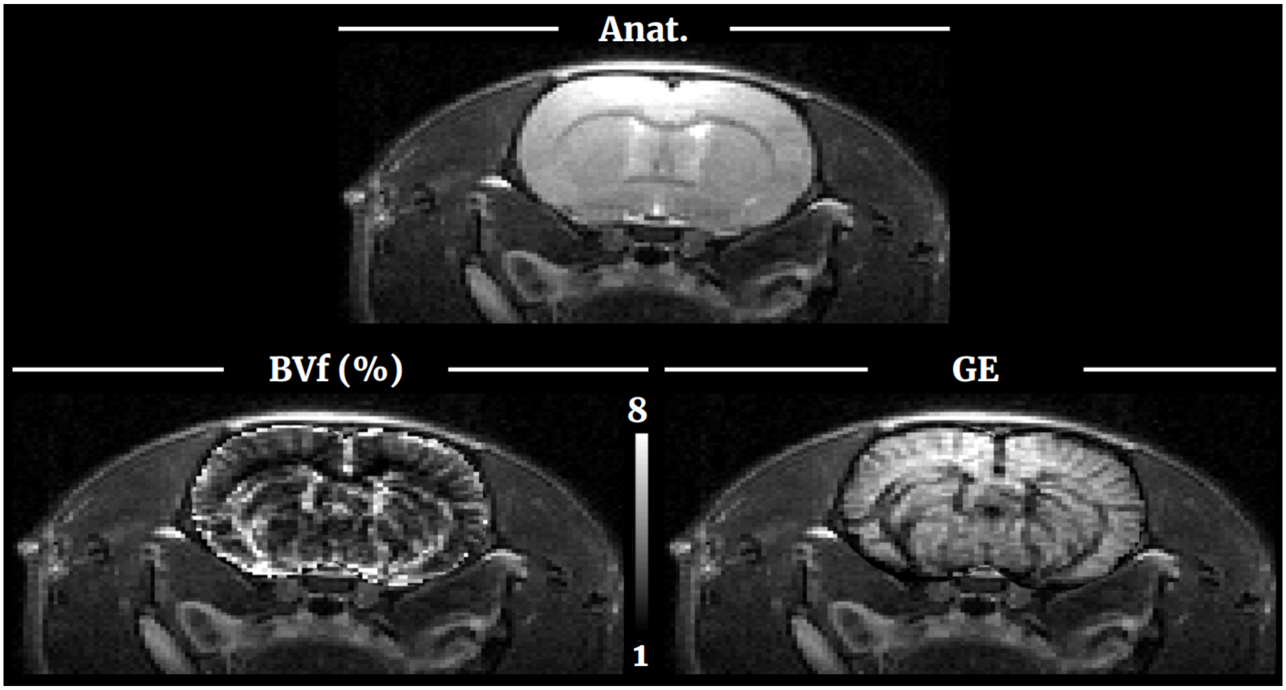

Sup. Fig. 3: BVf map and 5th gradient echo (GE) from the post-CA GESFIDSE (TE = 16.2 ms), overlayed on the analytical image, for one healthy animal. The signal attenuation from the CA makes the larger vascular structures well visible on the GE image. They correspond to regions where the BVf estimates are higher.

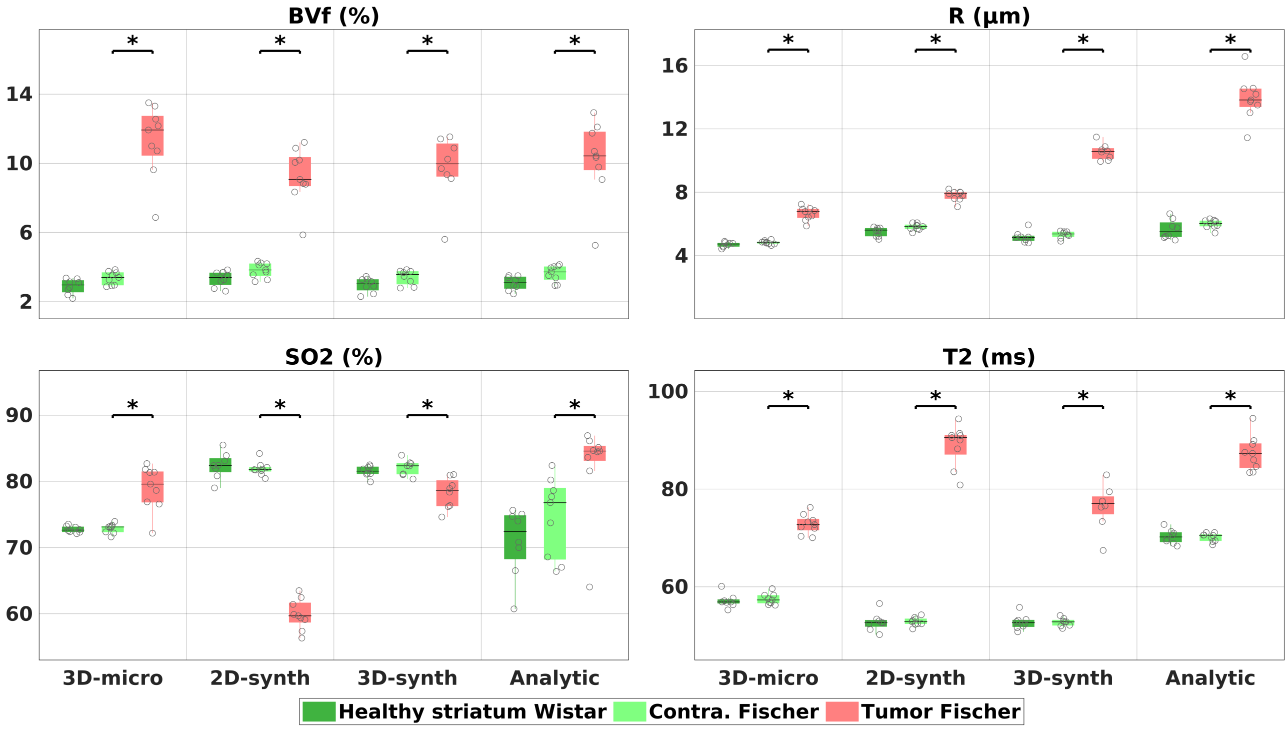

Sup. Fig. 4: Quantitative estimates of the 4 parameters for the different methods. All results from MRvF were obtained with the DBM method. "Healthy" values are averaged in the striatum for each animal, "Tumor" values from the lesion, and "Contra." from a contralateral ROI matching the tumor's location and size. One dot corresponds to one ROI in one animal. Stars indicate the significance with a p-value $\leq$ 5% (Tumor vs Contra).

BVf		3D-micro			2D-Synth			3D-synth			Analytic		
		Control	Healthy	Tumor	Control	Healthy	Tumor	Control	Healthy	Tumor	Control	Healthy	Tumor
3D-micro	Control		0.069	<0.001	0.153	0.001	<0.001	0.669	0.118	<0.001	0.935	0.023	<0.001
	Healthy			<0.001	0.794	0.028	<0.001	0.020	0.771	<0.001	0.056	0.380	<0.001
	Tumor				<0.001	<0.001	0.005	<0.001	<0.001	0.003	<0.001	<0.001	0.009
2D-Synth	Control					0.032	<0.001	0.060	0.997	<0.001	0.139	0.317	<0.001
	Healthy						<0.001	<0.001	0.019	<0.001	0.001	0.231	<0.001
	Tumor							<0.001	<0.001	0.858	<0.001	<0.001	0.844
3D-synth	Control								0.040	<0.001	0.580	0.007	<0.001
	Healthy									<0.001	0.105	0.272	<0.001
	Tumor										<0.001	<0.001	0.705
Analytic	Control											0.018	<0.001
	Healthy												<0.001
	Tumor												

Table 1: p-values for crossed 2-sample t-test on DBL BVf results. color change at p=0.05

Sup. Table 1

R		3D-micro			2D-Synth			3D-synth			Analytic		
		Control	Healthy	Tumor	Control	Healthy	Tumor	Control	Healthy	Tumor	Control	Healthy	Tumor
3D-micro	Control		0.027	<0.001	<0.001	<0.001	<0.001	0.781	0.001	<0.001	0.016	<0.001	<0.001
	Healthy			<0.001	<0.001	<0.001	<0.001	0.251	0.011	<0.001	0.054	<0.001	<0.001
	Tumor				<0.001	<0.001	<0.001	<0.001	<0.001	<0.001	<0.001	<0.001	<0.001
2D-Synth	Control					0.008	<0.001	0.005	0.169	<0.001	0.578	0.168	<0.001
	Healthy						<0.001	<0.001	<0.001	<0.001	0.026	0.133	<0.001
	Tumor							<0.001	<0.001	<0.001	<0.001	<0.001	<0.001
3D-synth	Control								0.025	<0.001	0.043	<0.001	<0.001
	Healthy									<0.001	0.701	0.008	<0.001
	Tumor										<0.001	<0.001	0.016
Analytic	Control											0.143	<0.001
	Healthy												<0.001
	Tumor												

Table 2: p-values for crossed 2-sample t-test on DBL R results. color change at p=0.05

Sup. Table 2

SO2		3D-micro			2D-Synth			3D-synth			Analytic		
		Control	Healthy	Tumor	Control	Healthy	Tumor	Control	Healthy	Tumor	Control	Healthy	Tumor
3D-micro	Control		0.392	<0.001	<0.001	<0.001	<0.001	<0.001	<0.001	<0.001	0.116	0.781	0.004
	Healthy			<0.001	<0.001	<0.001	<0.001	<0.001	<0.001	<0.001	0.126	0.673	0.002
	Tumor				0.935	0.438	<0.001	0.168	0.094	0.039	<0.001	0.010	0.661
2D-Synth	Control					0.418	<0.001	0.073	0.037	0.031	<0.001	0.012	0.637
	Healthy						<0.001	<0.001	<0.001	0.005	<0.001	0.013	0.399
	Tumor							<0.001	<0.001	<0.001	0.003	<0.001	<0.001
3D-synth	Control								0.478	0.149	<0.001	0.001	0.837
	Healthy									0.195	<0.001	0.001	0.720
	Tumor										<0.001	0.001	0.309
Analytic	Control											0.198	0.002
	Healthy												0.023
	Tumor												

Table 3: p-values for crossed 2-sample t-test on DBL SO2 results. color change at p=0.05

Sup. Table 3

T2		3D-micro			2D-Synth			3D-synth			Analytic		
		Control	Healthy	Tumor	Control	Healthy	Tumor	Control	Healthy	Tumor	Control	Healthy	Tumor
3D-micro	Control		0.697	<0.001	<0.001	<0.001	<0.001	<0.001	<0.001	<0.001	<0.001	<0.001	<0.001
	Healthy			<0.001	<0.001	<0.001	<0.001	<0.001	<0.001	<0.001	<0.001	<0.001	<0.001
	Tumor				<0.001	<0.001	<0.001	<0.001	<0.001	0.170	0.011	0.005	<0.001
2D-Synth	Control					0.951	<0.001	0.311	0.121	<0.001	<0.001	<0.001	<0.001
	Healthy						<0.001	0.114	0.005	<0.001	<0.001	<0.001	<0.001
	Tumor							<0.001	<0.001	<0.001	<0.001	<0.001	0.284
3D-synth	Control								0.618	<0.001	<0.001	<0.001	<0.001
	Healthy									<0.001	<0.001	<0.001	<0.001
	Tumor										0.263	0.188	<0.001
Analytic	Control											0.827	<0.001
	Healthy												<0.001
	Tumor												

Table 4: p-values for crossed 2-sample t-test on DBL T2 results. color change at p=0.05

Sup. Table 4

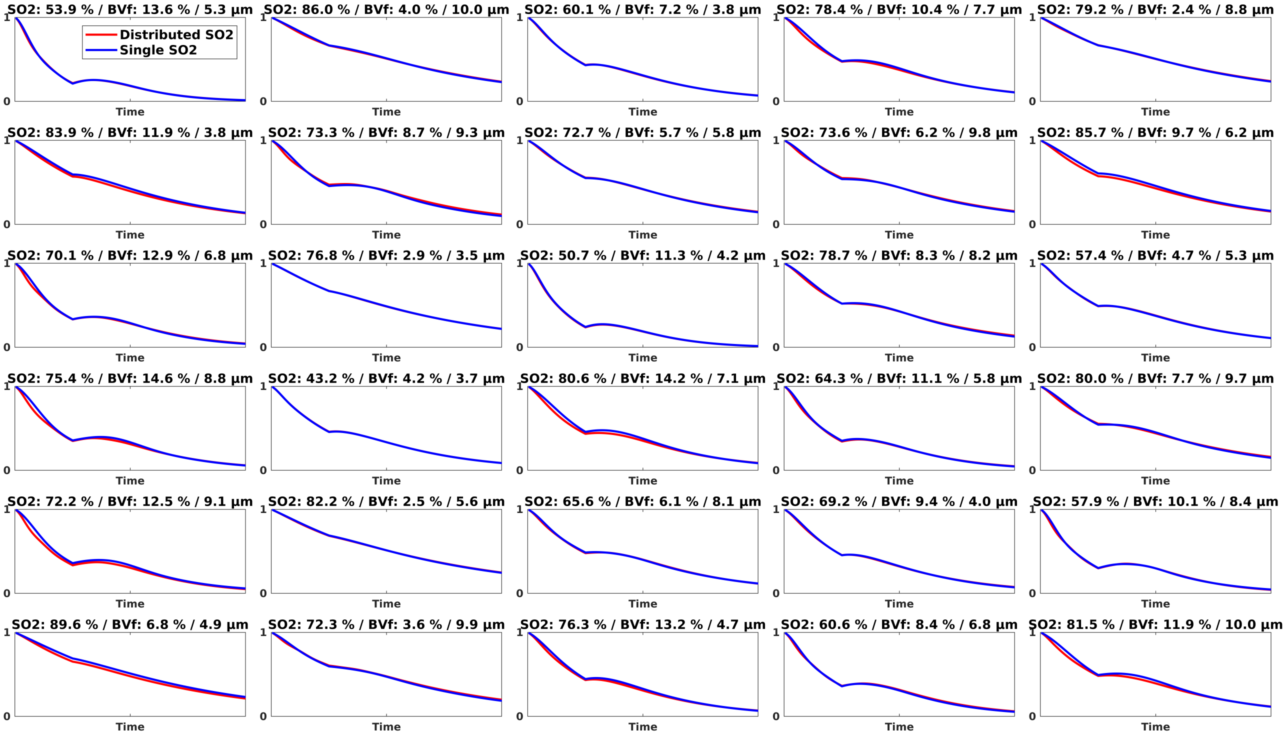

Sup. Fig. 5: Comparison of simulated signals under two different models. The red curves correspond to 3D synthetic voxels in which individual vessels have different SO_2 values, see examples on Sup. Fig. 6. The blue curves were obtained by using the same geometries but assigning a single SO_2 value to the whole vascular network, corresponding to the mean of the distribution used for the red curves.

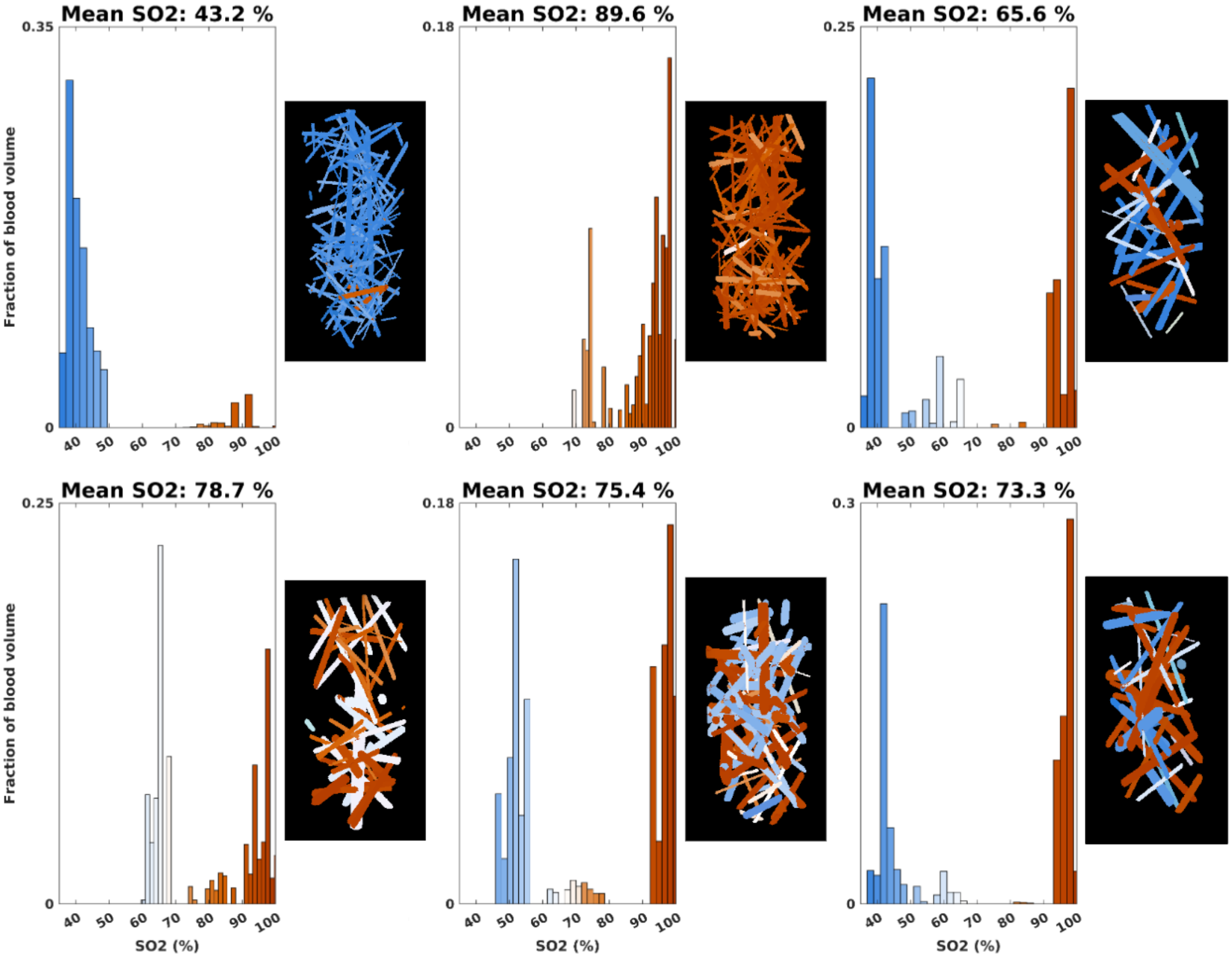

Sup. Fig. 6: Examples of voxels and their SO_2 distributions used to obtain the "Distributed SO2" curves on Sup. Fig. 5. The bar charts represent the distribution of SO2 across the sub-voxels elements that constitute the vascular network. The mean SO_2 across these sub-voxel elements is computed and used to obtain the "Single SO2" curves on Sup. Fig. 5.
